# Development of GABAergic Interneurons in the Human Cerebral Cortex

**DOI:** 10.1111/ejn.70136

**Published:** 2025-05-12

**Authors:** Oscar Marín

**Affiliations:** ^1^ Centre for Developmental Neurobiology, Institute of Psychiatry, Psychology and Neuroscience King's College London London UK; ^2^ Medical Research Council Centre for Neurodevelopmental Disorders King's College London London UK

**Keywords:** cerebral cortex, development, ganglionic eminences, human, interneuron, pallium, subpallium

## Abstract

GABAergic interneurons are critical regulators of information processing in the cerebral cortex. They constitute a heterogeneous group of neurons with unique spatial and temporal capabilities to control information flow and influence neural network dynamics through inhibitory and disinhibitory mechanisms. Interneuron diversity is largely conserved between rodents and primates, which indicates that the addition of new types of GABAergic neurons is not the most critical innovation of the primate cortex. In contrast, interneurons are much more abundant and seem more widely interconnected in the cerebral cortex of primates than in rodents, suggesting selective evolutionary pressure in the mechanisms regulating the generation, survival and maturation of cortical interneurons. Recent studies are beginning to shed light on the cellular and molecular mechanisms controlling the development of cortical interneurons in humans, from their generation in the embryonic telencephalon to their early integration in cortical networks. These studies identified many features in the development of human cortical interneurons that are shared with other mammals, along with distinctive features that seem characteristic of the primate brain, such as a previously unrecognised protracted period of neurogenesis and migration that extends the earliest stages of interneuron development into the first months of postnatal life in humans.

## Graphical abstract

Recent research is starting to shed light on the cellular and molecular processes that regulate the development of cortical GABAergic interneurons in humans, tracing their formation in the foetal telencephalon to their initial integration into neuronal networks in the postnatal cerebral cortex. This review outlines our present knowledge of this process.

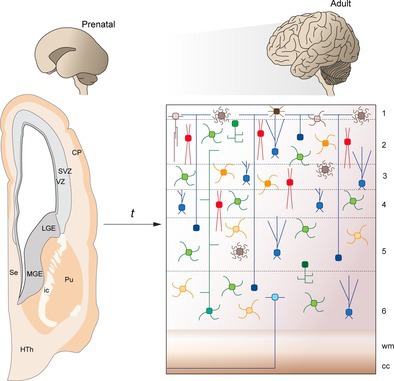

## 1 Introduction

GABAergic interneurons are crucial for the control of inhibition in the cerebral cortex. They control the flow of information in cortical networks, normalise the activity of excitatory neurons and perform crucial computational functions (Kepecs and Fishell 2014). More broadly, interneurons are critical in controlling the timing of neuronal activity and the synchronisation of cortical networks (Buzsáki and Draguhn 2004; Klausberger and Somogyi 2008). The astonishing diversity of cortical GABAergic interneurons with distinct connectivity profiles is thought to have increased the computational power of cortical circuits during evolution (Tremblay et al. 2016; Wang et al. 2004).

**FIGURE 1:**
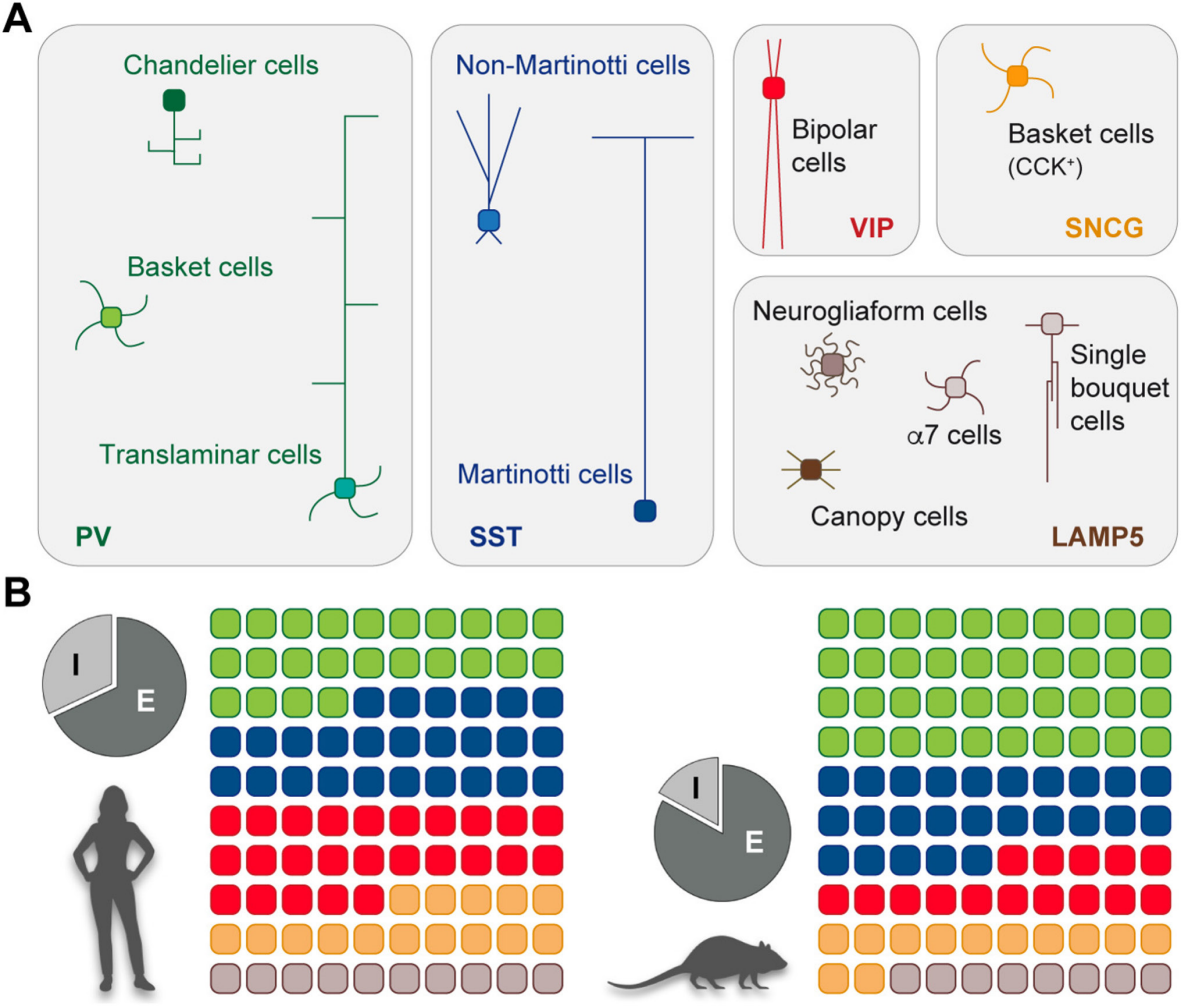
Cortical interneurons in rodents and humans. (A) A combination of morphological, biochemical, intrinsic and connectivity properties distinguishes different interneuron populations. There are five main subclasses of cortical interneurons, largely conserved between rodents and primates, including humans. (B) The relative proportion of cortical interneurons in the human cortex is much larger than in rodents. In addition, the contribution of CGE‐derived cells to the complement of cortical interneurons is more significant in primates than in rodents.

Recent transcriptomic studies suggest that the human cerebral cortex contains essentially the same types of GABAergic interneurons as other mammalian species, with a few exceptions. Cortical interneurons can be broadly subdivided into five large subclasses or groups: (1) parvalbumin‐expressing (PV^+^) fast‐spiking interneurons, including chandelier cells, basket cells and translaminar cells; (2) dendrite‐targeting interneurons expressing somatostatin (SST^+^), including Martinotti and non‐Martinotti cells; (3) a diverse range of bipolar cells expressing vasoactive intestinal peptide (VIP^+^); (4) large and small basket cells expressing cholecystokinin, often identified by the expression of synuclein gamma (SNCG^+^); and (5) a heterogeneous group of interneurons characterised by the expression of lysosomal associated membrane protein family member 5 (LAMP5^+^) that includes neurogliaform cells, single bouquet cells, canopy cells and α7 cells (Figure 1). Each subclass of interneurons comprises more refined subtypes of interneurons with unique morphological, electrophysiological and transcriptional properties. Importantly, there is a relatively high degree of divergence in cell type–specific gene expression across species, suggesting evolutionary specialisation among interneurons (BRAIN Initiative Cell Census Network (BICCN) 2021; Hodge et al. 2019). In other words, human interneurons most likely display species‐specific adaptations superimposed on conserved gene expression programmes.

Although cortical interneuron identities are broadly conserved between rodents and primates, including humans, some crucial differences have been identified (Figure 1). For example, analysis of three‐dimensional electron microscopy datasets from the neocortex of humans, macaques and mice revealed a 2.5‐fold increase of inhibitory interneurons from mouse to human (Loomba et al. 2022). Similarly, stereological analyses identified a higher proportion of calretinin‐expressing interneurons (frequently VIP^+^) in the human hippocampus than in rodents (Takács et al. 2025). These observations are consistent with recent single‐nucleus transcriptomic and spatial transcriptomic studies, which indicate that the ratio of cortical excitatory (E) to inhibitory (I) neurons in humans is close to 2:1 (Fang et al. 2022; Jorstad et al. 2023; Krienen et al. 2020), except in the primary visual cortex, where the cellular E/I ratio is roughly 5:1—a number comparable with the mouse neocortex E/I ratio (Meyer et al. 2011). Thus, except for the primary visual cortex (and perhaps other areas yet to be studied), the relative abundance of interneurons in the human cerebral cortex is much higher than in rodents.

There are also significant differences in the relative contributions of different subclasses of interneurons (Figure 1). For example, PV^+^ fast‐spiking interneurons are much more abundant than SST^+^ interneurons in most areas of the mouse neocortex (Rudy et al. 2011). However, the relative abundance of PV^+^ and SST^+^ interneurons is similar in macaques and humans (Chen et al. 2023; Jorstad et al. 2023). Other interneurons, including the VIP^+^ and LAMP5^+^ subclasses, are comparatively more abundant in primates, including humans, than in mice (Hodge et al. 2019; Krienen et al. 2020). In some specific cases, these differences are rather extreme. Neurogliaform cells expressing LAMP5 and the transcription factor LHX6 are rare in rodents but copious in the primate cerebral cortex (Krienen et al. 2020). In other cases, the observed differences may represent novel acquisitions of the primate lineage. For instance, Layer 1 interneurons seem exceptionally diverse in humans, and some, like rosehip cells, do not seem to have a homologous cell type in mice (Boldog et al. 2018; Chartrand et al. 2023).

The increased proportion of interneurons in the primate cerebral cortex compared with rodents and the changes in the relative abundance of different interneuron subtypes are likely rooted in development. In the following sections, I will review how variations in their generation, migration and maturation may have contributed to shaping the complement of GABAergic interneurons in the human cerebral cortex.

## 2 Origin of Cortical Interneurons in Humans

Since the seminal discovery by Anderson and colleagues of the subpallial origin of cortical GABAergic interneurons in mice (Anderson et al. 1997), numerous studies have confirmed and extended these observations. Cortical interneurons in rodents originate from progenitor cells in different regions of the subpallium, most notably the medial and caudal ganglionic eminences (MGE and CGE, respectively) and, to a lesser extent, the lateral ganglionic eminence (LGE) and the preoptic region (Lim et al. 2018; Wamsley and Fishell 2017). Multiple lines of evidence demonstrate that most cortical interneurons in humans are also produced in the ganglionic eminences. However, an increasing number of studies suggest a pallial origin for a yet‐to‐be‐characterised population of GABAergic cells.

### 2.1 Generation of Cortical Interneurons in the Subpallium

Analysis of the expression of key transcription factors indicates that the fundamental molecular organisation of the human subpallium is similar to that described in other mammals (Casalia et al. 2021; Flames et al. 2007; Ma et al. 2013). Several progenitor domains can be distinguished within the human ganglionic eminences, characterised by the expression of conserved transcription factors like NKX2‐1, PAX6, GSX2 and NR2F2 (Alzu’bi et al. 2017; Hansen et al. 2013; Ma et al. 2013; Pauly et al. 2014). Although current studies lack the resolution of similar work in rodents, the human subpallium likely contains segregated progenitor pools that generate different GABAergic interneuron subtypes. In addition, interneurons in the developing human cortex express many of the same markers previously identified in GABAergic interneurons tangentially migrating from the subpallium in rodents, such as LHX6, SOX6, NR2F2 and SP8 (Hansen et al. 2013; Ma et al. 2013). These observations reinforce the notion that most cortical interneurons are generated in the embryonic subpallium in humans.

Recent single‐cell RNA sequencing (scRNA‐seq) studies have more comprehensively characterised the molecular programmes of GABAergic interneurons in the human ganglionic eminences than earlier histological analyses. Their findings suggest that mice and humans essentially share molecular hierarchies and gene regulatory networks governing the early specification of cortical interneurons (Feng et al. 2025; Shi et al. 2021; Velmeshev et al. 2023; Yu et al. 2021; Zhao et al. 2022). Using data integration approaches across different developmental stages, these studies also revealed that newborn neurons in the foetal subpallium are already specified to acquire the fates of cortical interneurons (Shi et al. 2021; Velmeshev et al. 2023). These studies reinforce the view that most human cortical interneurons originate from the MGE and CGE, like in rodents.

### 2.2 Generation of Cortical Interneurons in the Pallium

Early studies exploring the origin of cortical interneurons in humans identified progenitor cells dividing in the pallium that give rise to neurons expressing GABA and DLX2 (Letinic et al. 2002). This conclusion was primarily based on short‐term retroviral lineage tracing experiments in organotypic slices, which could not exclude the possibility that the progenitor cells originated in the subpallium and continued to divide locally, as shown in rodents for some CGE‐derived interneurons (Magueresse et al. 2011; Riccio et al. 2011). Consistent with this notion, a subsequent study using comparable methods only identified a handful of DLX2^+^ progenitors in the pallium, and they were systematically close to the LGE and CGE (Hansen et al. 2013). Furthermore, some olfactory bulb interneurons are produced from pallial progenitors in both rodents and humans (Cai et al. 2013; Delgado et al. 2022; Fuentealba et al. 2015; Kohwi et al. 2007; Young et al. 2007) and can be potentially mistaken for cortical interneurons without the appropriate controls.

Nkx2‐1 is required for the specification of the MGE (Sussel et al. 1999), but it is rapidly downregulated in GABAergic interneurons migrating to the cerebral cortex in mice (Marín et al. 2001; Nóbrega‐Pereira et al. 2008). NKX2‐1^+^ cells seem absent from the human pallium before 13 postconception weeks (Hansen et al. 2013; Ma et al. 2013; Pauly et al. 2014). In contrast, other studies identified a small contingent of NKX2‐1^+^ cells in the human pallium during the second and third gestational trimesters (Alzu’bi et al. 2017; Arshad et al. 2016; Jakovcevski et al. 2011; Radonjić et al. 2014), some of which seemed to undergo active proliferation (Jakovcevski et al. 2011; Radonjić et al. 2014). This has been taken as an indication that at least some cortical interneurons are generated in the human pallium. However, these cells may also derive from the MGE but continue to divide in the pallium. Furthermore, considering the substantial changes in gene expression observed among homologous cortical neurons between mice and humans (BRAIN Initiative Cell Census Network (BICCN) 2021; Hodge et al. 2019), it is also conceivable that some interneuron subtypes generated in the subpallium retain expression of NKX2‐1 in the human cortex.

**FIGURE 2:**
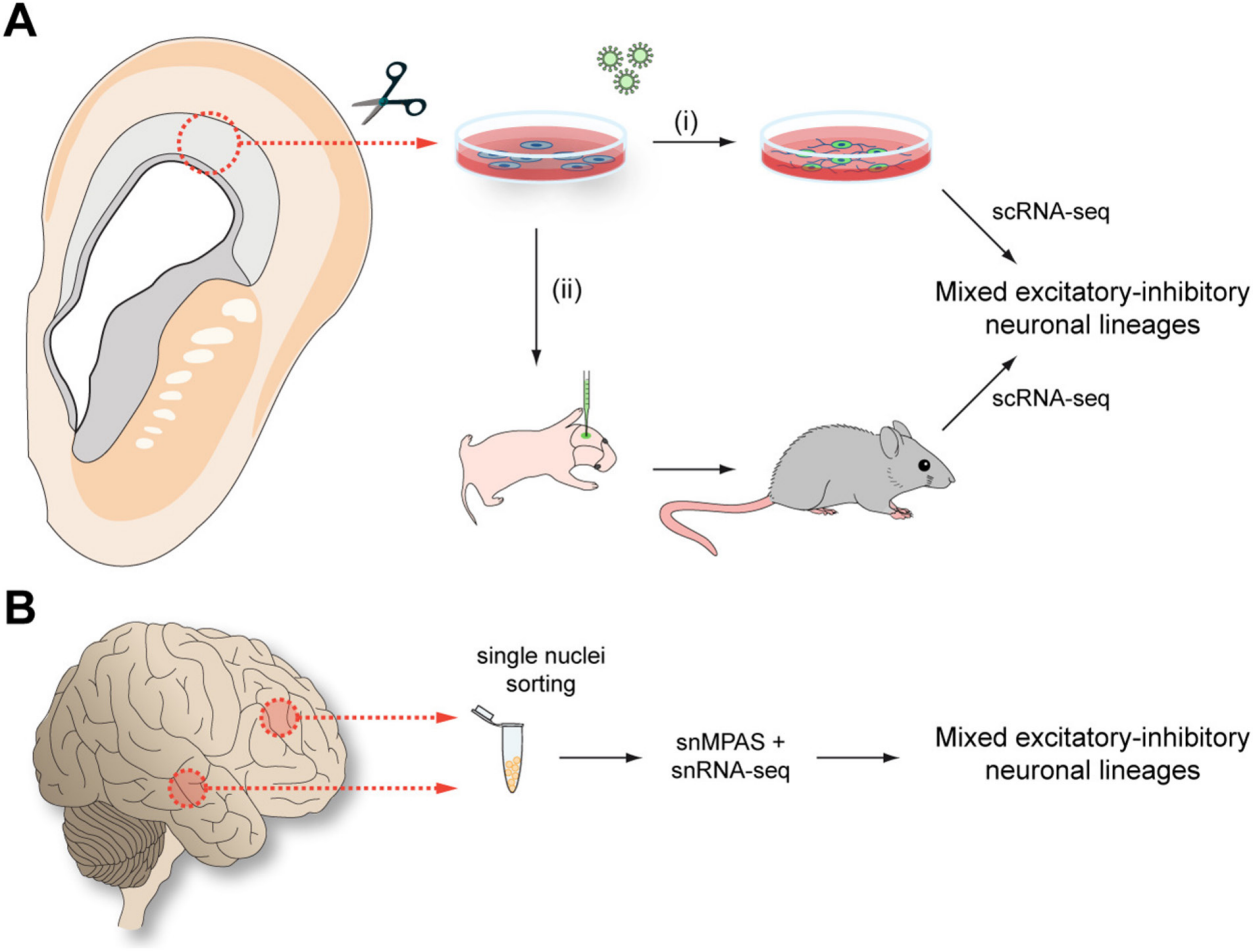
Recent experimental evidence on the origin of cortical interneurons in the human pallium. (A) Cells were isolated from the progenitor zone of the embryonic human pallium at postconception Weeks 13 and 16 and infected with lentiviruses expressing a molecular barcode library. These cells were then either (i) cultured for 6 weeks before analysis or (ii) transplanted into the cortex of newborn immunocompromised mice and allowed to develop for 6 weeks before analysis. In both cases, scRNA‐seq analysis of the labelled cells revealed mixed clones containing excitatory and inhibitory neurons (Delgado et al. 2022). (B) Single nuclei were isolated from the frontal and temporal cortices of an adult brain. The DNA and RNA of individual nuclei were analysed simultaneously using ultra‐deep massive parallel amplification (MPAS) sequencing and snRNA‐seq. The results of these analyses identified mixed clones containing excitatory and inhibitory neurons (Chung et al. 2024).

Although the notion that human cortical interneurons may also derive from pallial progenitors remains contentious, recent studies have further supported this idea (Figure 2). For example, clonal lineage tracing experiments in which neuronal fate was assessed using scRNA‐seq revealed that second‐trimester human pallial progenitors can generate both excitatory and inhibitory neurons in vitro and following their xenotransplantation into the neonatal cortex of mice (Delgado et al. 2022). Some of these progenitors may correspond to intermediate progenitors simultaneously expressing DLX5 and EOMES (TBR2) in the human pallium (Pebworth et al. 2021). Moreover, treatment of outer/basal radial glial cells isolated from the human pallium with leukaemia‐inhibitory factor stimulates the production of GABAergic neurons in vitro, with the resulting cells acquiring a fate like CGE‐derived interneurons (Andrews et al. 2023). This is consistent with previous observations suggesting that cortical progenitors can support the generation of GABAergic neurons in vitro, both in rodents and humans (Alzu'bi et al. 2017; Götz and Bolz 1994; He et al. 2001). However, a caveat of these experiments is the observation that certain factors added to the culture medium, such as FGF2, may artificially promote GABAergic fate (Gabay et al. 2003).

Although doubts remain about whether the observed lineage relationship between cortical excitatory and inhibitory neurons occurs in vivo, two independent studies have strengthened this possibility. Using brain mosaic variation within specific cell types to reflect clonal dynamics, they identified lineages containing inhibitory and excitatory neurons within the same cortical lobe (Chung et al. 2024; Kim et al. 2023). The most logical interpretation of these findings is that pallial progenitors can produce both classes of neurons. However, these studies cannot establish when these common lineages originate, and they could potentially predate the patterning of the telencephalon into pallial and subpallial compartments. A small sample size and other technical shortcomings also limit these studies. Still, they provide the most direct evidence for a pallial origin of a significant fraction of cortical interneurons to date.

## 3 Interneuron Neurogenesis

The large density of interneurons in the human cerebral cortex is linked to profound changes in the generation of these cells. During human foetal development, the ganglionic eminences have massively expanded the pool of subventricular zone (SVZ) progenitor cells, adopted distinctive patterns of progenitor cell organisation and clustering and extended the period of neurogenesis to nearly the end of pregnancy. These evolutionary adaptations likely underlie the extraordinary increase in the relative proportion of cortical interneurons characteristic of humans and other primates (Fang et al. 2022; Jorstad et al. 2023; Krienen et al. 2020; Loomba et al. 2022).

The human ganglionic eminences feature a massively enlarged SVZ that continues to expand well into the second trimester of gestation. As in rodents, this region contains many intermediate progenitor cells characterised by the expression of ASCL1 and DLX2 (Hansen et al. 2013). In addition, the SVZ also includes a small population of progenitors that seem to retain stem cell properties like radial glial cells (Hansen et al. 2013; Shi et al. 2021). These cells do not seem to have detectable radial fibres directed towards the mantle zone (Hansen et al. 2013), a feature of outer/basal radial glial cells in the developing pallium. Although the SVZ of the MGE acquires unique features from the early stages of neurogenesis, the SVZ of the LGE and CGE forms a morphological continuum without distinctive boundaries. Like rodents, the gradual transition in molecular markers such as NR2F2 defines the distinct progenitor pools, distinguishing both regions. Remarkably, the CGE contains a higher density of dividing cells than the MGE (Arshad et al. 2016; Hansen et al. 2013), which may account for the increased proportion of CGE‐derived interneurons in the human cerebral cortex.

**FIGURE 3:**
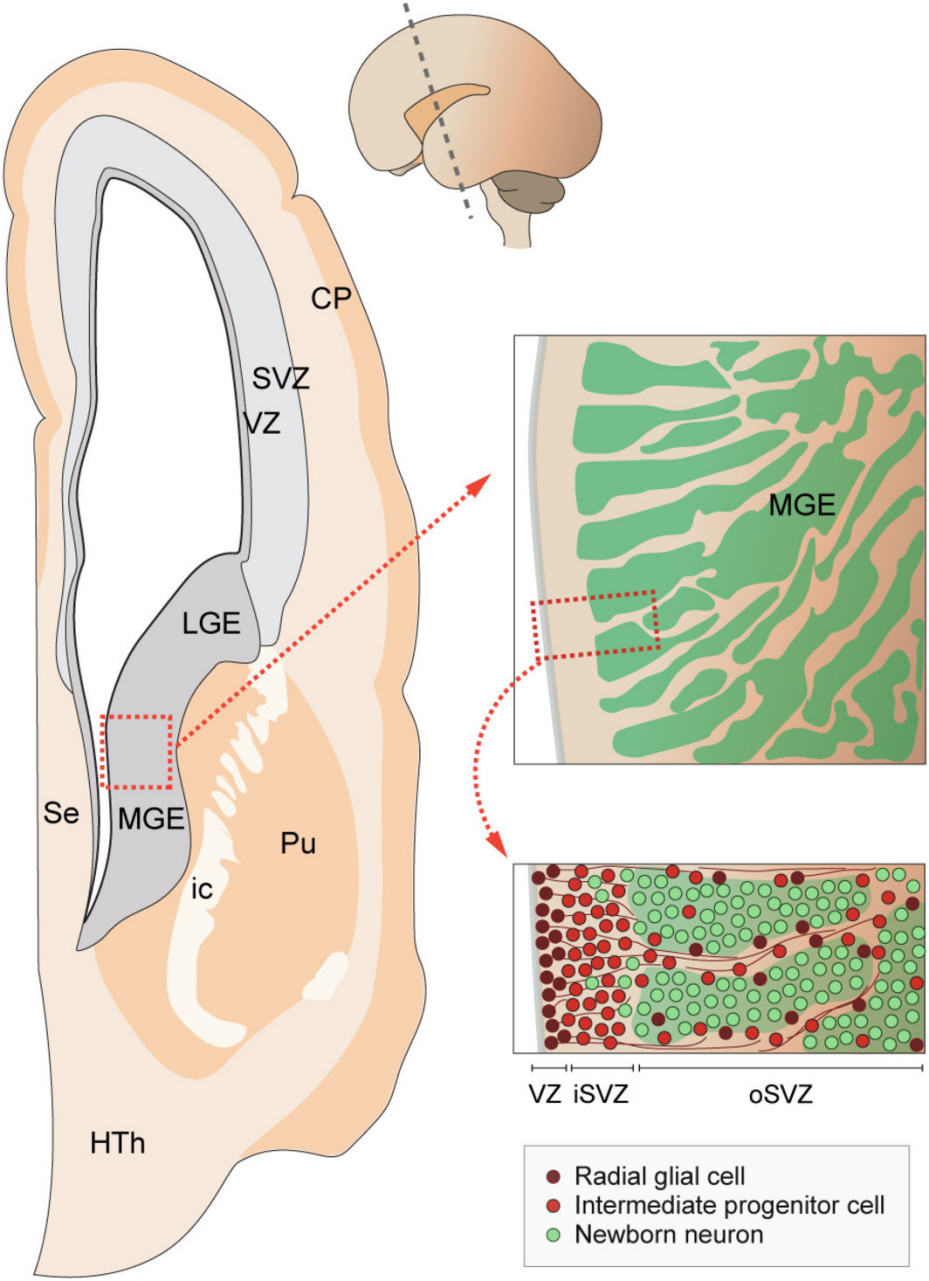
Organisation of the human medial ganglionic eminence (MGE). Radial glial cells line up the ventricular zone (VZ) immediately adjacent to a compact inner subventricular zone (iSVZ) comprising many intermediate progenitor cells. The MGE outer subventricular zone (oSVZ) is massively expanded at peak neurogenesis. In this region, newborn neurons (DCX^+^ and LHX6^+^) are organised into what seems a complex net of interconnected burrows. Progenitor cells, likely intermediate progenitor cells and outer radial glial cells, are also found within and around the clusters of newborn neurons. CP, cortical plate; HTh, hypothalamus; ic, internal capsule; LGE, lateral ganglionic eminence; Pu, putamen; Se, septum.

The human MGE exhibits a characteristic pattern of cell organisation (Figure 3). At the peak of neurogenesis, the MGE is traversed by thick bundles of fibres containing vimentin and nestin, two proteins expressed by radial glial cells. These fibres are decorated with progenitor cells, which form long streams linking the ventricular zone with the SVZ. The space between the nestin^+^ fibres is occupied by large clusters of cells expressing doublecortin (DCX^+^) and the transcription factor LHX6, among which proliferating cells intermingle and persist until birth (Hansen et al. 2013; Paredes et al. 2022). It is unclear how many different progenitor cells populate the MGE SVZ, but both SOX2^+^DCX^+^ and SOX2^+^DCX^−^ actively proliferating cells have been described (Paredes et al. 2022). Whether any of these cells have similar properties to those described in outer/basal radial glial cells in the human pallium remains to be elucidated. Interestingly, although similar progenitor cells have been found throughout the LGE and CGE SVZ, they are present in much smaller proportions. This observation suggests that the distinctive organisation of the human MGE might sustain the maintenance of a large pool of progenitor cells in the SVZ. Of note, the SVZ of the porcine MGE also contains clusters of neuroblasts resembling, on a much smaller scale, those described in humans and absent in mice (Casalia et al. 2021).

Finally, the time span of interneuron neurogenesis is another distinctive feature of humans. Although the density of progenitor cells is substantially reduced after the second trimester of pregnancy, cortical interneurons continue to be generated in the ganglionic eminences during the third trimester (Arshad et al. 2016). Remarkably, recent analyses revealed that some level of neurogenesis persists in the CGE after birth (Nascimento et al. 2024), contradicting the widely held view that human neurogenesis is complete by this time. Notably, some cortical interneurons may also be generated postnatally in rodents (Inta et al. 2008). The protracted neurogenesis in the ganglionic eminences likely contributes to the expanded complement of interneurons in the human cerebral cortex and has significant clinical implications.

## 4 Migration of Cortical Interneurons

Cortical interneurons in the human ganglionic eminences express receptors identical to those found to regulate the tangential migration of interneurons in rodents, including ERBB4 and CXCR4 (Marín 2013; Shi et al. 2021; Toudji et al. 2023). This indicates that the conserved traits in the development of interneurons are not limited to the initial specification of progenitor cells but also encompass crucial aspects of their migration and differentiation.

Because of the difficulties inherent in investigating interneuron migration in organotypic slice cultures derived from human foetal tissue (Letinic et al. 2002), our current understanding of the cellular and molecular mechanisms regulating the migration of cortical interneurons mainly stems from studies in three‐dimensional brain organoids. In particular, the development of methods to generate regionalised neural organoids with pallial or subpallial features and their subsequent fusion into assembloids has revolutionised our ability to model cortical interneuron migration (Bagley et al. 2017; Birey et al. 2017; Xiang et al. 2017). These studies revealed that human cortical interneurons adopt a saltatory mode of migration involving the extension and remodelling of branched leading processes followed by myosin‐dependent soma and nuclear translocation, as described in rodents (Martini et al. 2008; Moya and Valdeolmillos 2004). Pharmacological manipulations also suggested that migrating human interneurons respond to chemokine signalling mediated by CXCR4 (Bagley et al. 2017; Birey et al. 2017; Xiang et al. 2017), which in rodents is responsible for guiding the tangential dispersion of interneurons throughout the developing cortex (Li et al. 2008; López‐Bendito et al. 2008; Sánchez‐Alcañiz et al. 2011; Tiveron et al. 2006; Wang et al. 2011). In addition, modulation of several neurotransmitter receptors seems to influence the tangential migration of cortical interneurons, as first reported in rodents (Bajaj et al. 2021; Bortone and Polleux 2009; López‐Bendito et al. 2003; Luhmann et al. 2015; Marín 2013). Taken together, these experiments indicate that the cellular and molecular mechanisms regulating the tangential migration of cortical interneurons might be generally conserved between rodents and primates, including humans.

Although it is widely assumed that neuronal migration in the developing cortex is largely completed during foetal development (Sidman and Rakic 1973), GABAergic interneurons continue to migrate into the human frontal and temporal lobes for several months after birth (Nascimento et al. 2024; Paredes et al. 2016; Sanai et al. 2011). These neurons seem to disperse tangentially through the SVZ both rostrally, into the frontal lobe, and caudally, into the entorhinal cortex and adjacent regions (Nascimento et al. 2024; Paredes et al. 2016). Interneurons migrating into the frontal lobe during postnatal stages seem to derive from different regions of the subpallium, including the MGE, dorsal LGE and CGE (Paredes et al. 2016; Schmitz et al. 2022). Transcriptional analyses have led to the suggestion that this migratory stream may contain neurons initially destined for the olfactory bulb but repurposed for the cerebral cortex because of the relative reduction in the size of the primate olfactory bulb (Schmitz et al. 2022). In contrast, most neurons migrating into the entorhinal cortex appear generated in the CGE and differentiate as neurogliaform cells (Nascimento et al. 2024). It is worth noting that the cellular and molecular mechanisms regulating the postnatal migration of interneurons might be substantially different from those controlling their initial tangential dispersion during the foetal period because the local environment these cells traverse is very different from the embryonic brain. Indeed, neurons en route towards the entorhinal cortex at postnatal stages move in chains and are surrounded by glial cells. These features are reminiscent of the rostral migratory stream through which adult‐born neurons reach the olfactory bulb in rodents (Lois et al. 1996).

The migratory stream that reaches the human frontal lobe in infants is reminiscent of a population of neurons that accumulate close to the anterior region of the SVZ in rodents at early postnatal stages and eventually seeds the medial prefrontal cortex with a late arrival pool of CGE‐derived interneurons (Magueresse et al. 2011; Riccio et al. 2011). Similarly, a caudal migratory stream linking the CGE with parahippocampal structures during embryonic stages has also been described in mice (Yozu et al. 2005). These observations suggest that these migratory streams might be evolutionary conserved among mammals but have been expanded in humans because of the protracted development of the human cerebral cortex. The fact that the migration of interneurons continues until the age of 2–3 years in humans in some cortical regions has critical implications for the assembly and refinement of neuronal circuits in these structures.

## 5 Maturation and Wiring

Our understanding of the events regulating the development of human interneurons after they arrive at their destination in the cerebral cortex is very rudimentary. For example, programmed cell death extensively prunes the final number of cortical interneurons in the mouse cortex during early postnatal stages (Wong and Marín 2019). However, we do not know whether a similar process occurs in humans. In mice, programmed cell death happens between postnatal Days 5 and 10, roughly corresponding to the perinatal period in humans. Although no study has estimated human interneuron numbers precisely, stereological analyses suggest that the total neuronal population of the human cortex decreases during late gestation, with no further changes after birth (Kjær et al. 2017; Larsen et al. 2006; Rabinowicz et al. 1996). These observations suggest that the programmed cell death of cortical interneurons, if existent in humans, likely occurs during the third trimester of gestation. Nevertheless, the protracted period of interneuron migration observed in some cortical regions (Nascimento et al. 2024; Paredes et al. 2016; Sanai et al. 2011) may delay this process to postnatal stages.

Connectomics studies have revealed that interneuron–interneuron connectivity might have expanded 10‐fold in primates, including humans (Loomba et al. 2022). However, few studies have explored the early wiring of cortical interneurons in humans. Analysis of single‐nucleus RNA sequencing datasets from the foetal and early postnatal cortex suggests that cortical interneurons mature by the end of the second trimester (Velmeshev et al. 2023), but this is unlikely the case for all interneuron populations considering that the neurogenesis of many of these cells extends until birth. Consistently, PV immunoreactivity, a feature of mature fast‐spiking basket cells, is relatively sparse during the first months of postnatal life and only seems to reach stable levels around year 8 in humans (Ábrahám et al. 2023; Grateron et al. 2003; Rogers et al. 2018), suggesting that at least some subclasses of cortical interneurons have a protracted maturation in humans. Similarly, recent efforts have begun to characterise the molecular composition of inhibitory synapses in the developing human cerebral cortex (Wang et al. 2023). Moreover, cortical assembloids are a promising model for investigating inhibitory connectivity in vitro (Bagley et al. 2017; Birey et al. 2017; Samarasinghe et al. 2021; Xiang et al. 2017), but it is presently unclear whether synaptic connectivity in organoids develops at the same rate and with the same specificity as in vivo. Overall, the mechanisms regulating the formation of cortical inhibitory synapses in humans remain largely unexplored.

## 6 Implications for Neurodevelopmental Disorders

Cortical interneuron dysfunction has been extensively linked to the pathology of neurodevelopmental disorders (Contractor et al. 2021; Dienel and Lewis 2019; Marín 2012, 2024), with synaptic connectivity defects as the suspected culprit for their involvement in pathophysiology. However, the recent findings of the protracted development of cortical interneurons in humans, including extensive neurogenesis in the third trimester of gestation and prominent neuronal migration during the first postnatal months, have significant clinical implications. For example, it has been suggested that a population of quiescent CGE progenitors from mid‐gestational stages gives rise to tumours and tubers in the cerebral cortex of patients with tuberous sclerosis (Eichmüller et al. 2022). Although this conclusion is primarily derived from experiments with cerebral organoids and remains to be validated in more patients, it reveals that the extended generation of cortical interneurons in humans is a disease vulnerability. Third‐trimester complications, including hypoxia‐ischemia, chorioamnionitis and haemorrhages, may also acutely impact the development of cortical interneurons, most notably those directed to the frontal and temporal lobes. For example, prematurity significantly increases the risk of germinal matrix haemorrhage due to the rupture of blood vessels in the developing ganglionic eminences (Chen et al. 2024), and premature babies that survive a germinal matrix haemorrhage are much more likely to develop a neurodevelopmental condition (Inder et al. 2023). In summary, the ongoing development of interneurons in the perinatal period highlights the importance of understanding how complications during these stages impact cortical function and how these effects can be mitigated.

## Author Contributions

Oscar Marín wrote this manuscript and prepared the figures.

## Conflicts of Interest

The author declares no conflicts of interest.

### Peer Review

The peer review history for this article is available at https://www.webofscience.com/api/gateway/wos/peer‐review/10.1111/ejn.70136.

## Data Availability

The authors have nothing to report.
